# Comparison of outcome measures and complication rates following three different approaches for primary total hip arthroplasty: a pragmatic randomised controlled trial

**DOI:** 10.1186/s13063-017-2368-7

**Published:** 2018-01-08

**Authors:** Adrian J. Talia, Cassandra Coetzee, Oren Tirosh, Phong Tran

**Affiliations:** Department of Orthopaedics, Western Health, Gordon Street, Footscray, VIC 3011 Melbourne, Australia

**Keywords:** Osteoarthritis, Total hip arthroplasty, Randomised, Patient-reported outcome measures, Surgical approach

## Abstract

**Background:**

Total hip arthroplasty is one of the most commonly performed surgical procedures worldwide. There are a number of surgical approaches for total hip arthroplasty and no high-level evidence supporting one approach over the other. Each approach has its unique benefits and drawbacks. This trial aims to directly compare the three most common surgical approaches for total hip arthroplasty.

**Methods/design:**

This is a single-centre study conducted at Western Health, Melbourne, Australia; a large metropolitan centre. It is a pragmatic, parallel three-arm, randomised controlled trial. Sample size will be 243 participants (81 in each group). Randomisation will be secure, web-based and managed by an independent statistician. Patients and research team will be blinded pre-operatively, but not post-operatively.

Intervention will be either direct anterior, lateral or posterior approach for total hip arthroplasty, and the three arms will be directly compared. Participants will be aged over 18 years, able to provide informed consent and recruited from our outpatients. Patients who are having revision surgery or have indications for hip replacement other than osteoarthritis (i.e., fracture, malignancy, development dysplasia) will be excluded from the trial.

The Oxford Hip Score will be determined for patients pre-operatively and 6 weeks, 6, 12 and 24 months post-operatively. The Oxford Hip Score at 24 months will be the primary outcome measure. Secondary outcome measures will be dislocation, infection, intraoperative and peri-prosthetic fracture rate, length of hospital stay and pain level, reported using a visual analogue scale.

**Discussion:**

Many studies have evaluated approaches for total hip arthroplasty and arthroplasty registries worldwide are now collecting this data. However no study to date has compared these three common approaches directly in a randomised fashion. No trial has used patient-reported outcome measures to evaluate success. This pragmatic study aims to identify differences in patient perception of total hip arthroplasty depending on surgical approach.

**Trial registration:**

Australian New Zealand Clinical Trials Registry, ACTRN12617000272392. Registered on 22 February 2017.

**Electronic supplementary material:**

The online version of this article (10.1186/s13063-017-2368-7) contains supplementary material, which is available to authorized users.

## Background

Total hip arthroplasty for osteoarthritis has long been demonstrated to be a cost-effective treatment for osteoarthritis of the hip with improvements in pain, improved function and quality of life [1–3]. Many studies have demonstrated the effectiveness of total hip arthroplasty as an intervention in terms of patient-reported outcome measures [4, 5]. Osteoarthritis is the condition with the single largest expenditure of the Australian health budget, with $1.64 billion spent on osteoarthritis in 2008–09, 77% of which was for surgical procedures relating to osteoarthritis [6].

Total hip arthroplasty is the most common operative intervention for the treatment of severe osteoarthritis, with 32,306 primary total hip arthroplasties reported in 2014, 88.5% of which were for osteoarthritis. The Australian Orthopaedic Association National Joint Replacement Registry reports that this rate is increasing, with a 5.4% increase in the annual amount of total hip replacements in 2014 compared with the previous 12 months [7]. Studies in the USA have estimated an almost 200% increase in demand for total hip arthroplasty on current rates by 2030 [8].

There are a number of different surgical approaches to total hip arthroplasty, with the most common ones performed including the direct lateral (Hardinge), posterior and direct anterior approaches.

### Posterior approach

This approach is the most common approach for total hip arthroplasty worldwide today, made popular by Moore in 1959 [9]. This approach allows excellent visualisation of the femoral shaft with reduced risk of femoral fracture but involves detachment of the short external rotators of the hip. Posterior capsulotomy is a theoretical risk for dislocation in this approach and the sciatic nerve is also at risk [10]. The abductor muscles are not incised; therefore, the incidence of gait disturbance (Trendelenburg gait) is reduced compared with the Hardinge approach. A Swedish joint registry study showed slightly better pain and functional scores for patient-reported outcome measures in patients who underwent a posterior approach compared with a direct lateral approach [11].

### Direct lateral approach

This was first described by Hardinge in 1982 [12]. This approach requires bisection of the anterior half of the periosteum overlying the greater trochanter and reflection of the gluteus medius and minimus muscles. The superior gluteal nerve and artery are at risk if the approach is extended proximally. The advantage of this approach is that it can be extended distally for greater exposure of the femur where necessary and theoretically has a lower dislocation risk than the posterior approach. Gluteal tendon splitting can lead to a post-operative Trendelenburg gait.

### Anterior approach

This approach to the hip joint was initially described by Hueter [13, 14] and later popularised by Smith-Petersen [15]. It utilises the anatomic internervous plane between the superior gluteal nerve laterally and femoral nerve medially and has recently been popularised for total hip replacement [16], but its first reported use for total hip replacement dates back to 1947 in France, by Judet and colleagues [17]. Studies have suggested higher rates of dislocation in patients who underwent a posterior approach for their total hip arthroplasty [18]. The neurovascular plane for the anterior approach is between the superior gluteal nerve and the femoral nerve. In some reports, patients who have undergone an anterior approach for total hip arthroplasty have shorter hospital stays than those who have undergone a posterior approach, and this is thought to be due to the muscle-sparing aspect of the approach [19, 20]. Compared with the posterior approach, the anterior approach involves less exposure of the femur for medullary reaming, and this may pose problems with regards to femoral component positioning and femoral shaft complications [21].

## Methods

### Experimental design

This is a randomised controlled trial conducted at one site, Western Health, Melbourne Australia. There will be 243 patients recruited across three trial arms. Consultant orthopaedic surgeons will be performing or directly supervising all surgery. The research design is a stratified block permuted randomisation and is non-blinded. We are expecting to recruit 243 patients in 24 months based on institutional case load. The total time of this study will be 5 years.

**Fig. 1:**
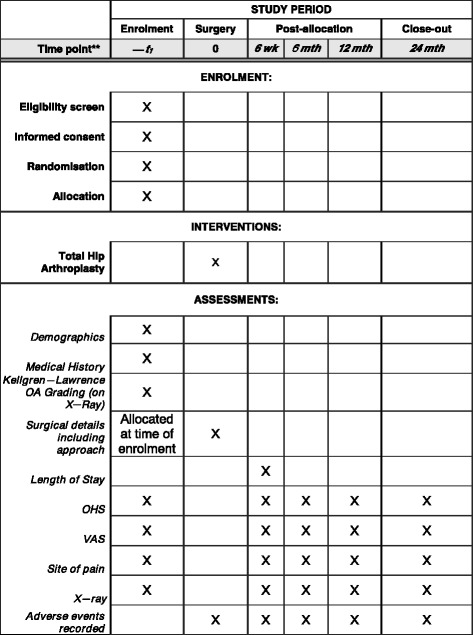
SPIRIT figure: trial timeline for recruitment, intervention, assessment and follow-up. OA, osteoarthritis; OHS, Oxford Hip Score; VAS, visual analogue scale

The trial will be registered, constructed and presented according to the recommendations of the CONSORT statement [22, 23], the trial protocol and manuscript has been prepared in accordance with the SPIRIT checklist (Additional file 1 and Fig. 1).

### Participants

Patients currently on the orthopaedic surgery waiting list at Western Health for a primary total hip arthroplasty will be invited to participate in the study. Patients referred to the clinic will be prospectively recruited prior to joining the waiting list and, once agreeable, will be randomised. Attendance at clinic is mandatory prior to surgery. Patients will undergo health assessment and provide informed consent to total hip arthroplasty. The department comprises consultant orthopaedic surgeons, orthopaedic registrars, resident medical officers and nursing and allied health staff. After initial screening, suitable patients will be a given a statement in plain English detailing the nature of the study and the commitment required. The consultant surgeon, research associates or resident medical officers will obtain informed consent for the study from suitable patients.

### Inclusion criteria

All adult patients (>18 years) who are on the waiting list for primary total hip arthroplasty will be considered eligible for the trial, unless they meet one of the exclusion criteria. This trial will include patients who have secondary osteoarthritis that could be post-traumatic, osteonecrosis or secondary to inflammatory arthropathy.

### Exclusion criteria

Patients who are having revision surgery or have indications for hip replacement other than osteoarthritis (i.e., fracture, malignancy, development dysplasia) will be excluded from the trial. Patients who are unable to complete the patient-reported outcome measures required, e.g., for mental health reasons or illiteracy, will be excluded from the trial.

### Intervention

Patients will be recruited from the Orthopaedic Outpatient Department at Western Health. Eligibility to participate will be assessed and patients will be asked to consent to enrol in the study. They will then be allocated to one of the three trial arms (anterior, lateral and posterior) and blinded to the allocation.

Operating surgeons will perform the approach that they are most familiar with using and the component system that they are most familiar with, to ensure that the study is not influenced by poor outcomes associated with surgeons performing approaches or components that they are not familiar with, e.g., Mr A, Mr B and Mr C will perform anterior approach total hip arthroplasty, Mr B, Mr D and Mr E will perform Hardinge approach total hip arthroplasty and Mr F, Mr G and Mr H will perform the posterior approach total hip arthroplasty. Nine surgeons will be involved in the study.

Pre-operatively the patients and the research team will be blinded to their allocation, whilst the treating surgeon will not be. However, blinding of the patients post-operatively will be impossible, owing to the nature of the incisions for the different approaches for hip arthroplasty.

### Standard of care

The components used will have the same design philosophy, with all patients receiving an uncemented acetabular component and anatomical shaped uncemented femoral stem or cemented femoral component. Sub-analysis will be performed between the cemented and uncemented stems. All patients will be subject to a standardised intraoperative and post-operative care protocol, including the use of tranexamic acid and local infiltration anaesthesia.

**Fig. 2:**
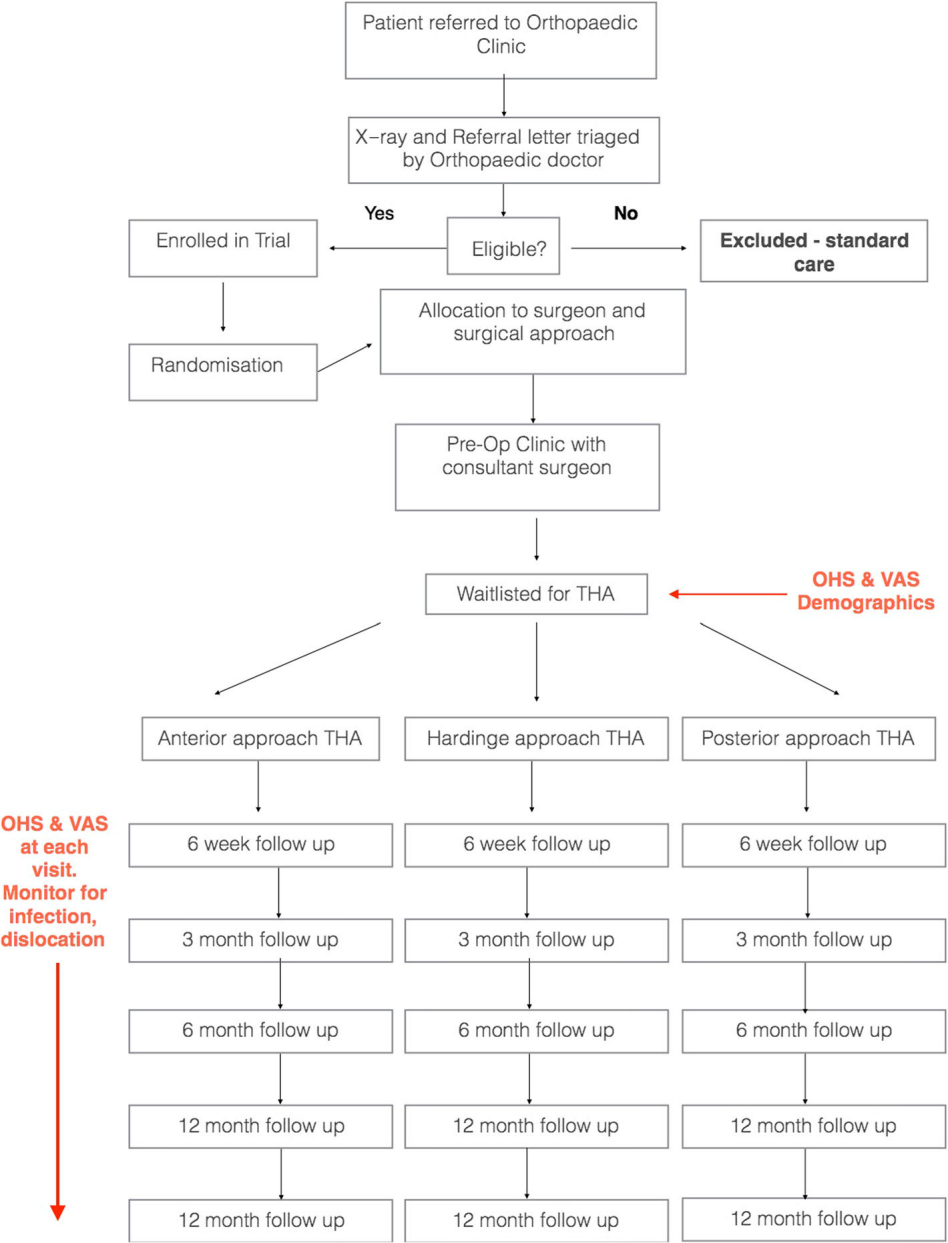
Trial flow pathway. OHS, Oxford Hip Score; THA, total hip arthroplasty; VAS, visual analogue scale

All patients will receive the standard routine post-operative management protocol of a wound review at 2 weeks, a further review with X-ray at 6 weeks and further information collected at 6, 12 and 24 months. Post-operative rehabilitation and physiotherapy will be standardised according to our institutional protocol and initiated for all patients at day 1 post-operatively (Fig. 2).

### Outcome assessments

#### Data capture

Data will be captured by the research associates and resident medical officers. All information on general medical, operative and device-related complications will be documented and tabulated utilising the secondary outcomes listed in this paper. Our clinic routinely collect patient-reported outcome measures; the collection of this information will not involve deviation from our standard of practice.

#### Primary outcome

The primary outcome measure for this study is the Oxford Hip Score; this will be recorded as shown in Fig. 1 at the following time points: pre-operatively and post-operatively at 6 weeks, 6, 12 and 24 months. The primary outcome measure for which our study is powered is the Oxford Hip Score at 24 months.

#### Secondary outcomes

Secondary outcome measures will be recorded as follows:

- Complications
  - ° Dislocation rates
  - ° Infection rates
  - ° Intraoperative and periprosthetic fracture rates
- Component position
  - ° Leg length discrepancy, offset, acetabular cup position
- Length of stay
- Visual analogue scale
  - ° If residual pain persists, the region of pain will be recorded as either buttock, trochanteric region or groin

#### Measurement tools

The measurement tools for this study are patient-reported outcome measures, specifically the Oxford Hip Score and a visual analogue scale for rating pain.

All information on general medical, operative and device-related complications will be documented and tabulated using the following clinical report forms:

- Demographics
- Past history
- Body mass index
- Medical comorbidities, including cardiorespiratory disease, malignancy, obesity, diabetes mellitus and smoking status
- Kellgren–Lawrence grade of osteoarthritis on plain radiographs
- Surgical approach
- Prosthesis used
- Complications or adverse event, specifically looking for:
  - ° Infection of hip joint
  - ° Dislocation of hip joint
  - ° Intraoperative fracture or periprosthetic fracture post-operatively
- Protocol deviation
- Study termination

All information on complications or adverse events (date of occurrence, description, severity, related to study device, treatment and resolution) will be recorded at the time of occurrence.

Collection of the outcome scores will be blinded, with the investigators or researchers involved in the collection of the scores blinded to the allocation.

### Timelines

This trial will be conducted over 5 years; we anticipate 3 years of recruitment and 2 years of follow-up for each patient. Figures 1 and 2 provide the time points at which patients will be seen and data recorded.

### Recruitment

All patients presenting to the Western Health outpatient clinic during the recruitment period and placed on the waiting list for primary total hip arthroplasty will be invited to participate in the study, providing that they do not meet any exclusion criteria. The senior author and principal investigator will oversee recruitment.

### Data management

The research information will be re-identifiable. This means that we will remove the participants’ names and give the research information a special code number. Only the research team can match the participants’ name to their code numbers, if it is necessary to do so.

All electronic data will be kept secure by being accessed on computer via password only. A nightly back-up performed on the computer will ensure all data are safely and securely stored. Any hardcopy of data will be identified by number only and kept secure in a locked filing cabinet. All information will be stored in the research office at Western Health. Only the named researchers will have access to the information.

In presentations or publications arising from this study, information will be provided in such a way that participants cannot be identified. Data will be presented as grouped data. All analysis is anticipated to be completed and published within 3 years of completing data collection.

### Statistical considerations

The null hypothesis for this study is that patient-reported outcomes following three different approaches for total hip arthroplasty are no different when directly compared 24 months post-procedure. In addition we hypothesise that complication rates are the same between approaches. There is no randomised controlled trial that directly compares these three approaches for total hip arthroplasty. We are hence designing our study as a superiority study, looking for a significant difference in Oxford Hip Score and visual analogue scale rating between the different trial arms.

#### Randomisation and enrolment

The randomisation list will be computer-generated by an independent statistician. A stratified block permuted randomisation will be used with the Kellgren–Lawrence grading scale (grade 0–2 vs. grade 3–4) as the stratification variable [24]. The block size will not be disclosed, to ensure concealment. Randomisation will be enabled through a secure, web-based application.

The surgeons performing the operation will be assigned to an operation type according to their expertise. Each surgeon will perform a single operation type and each operation type will be conducted by three surgeons.

A patient will be managed by one of the three surgeons corresponding to the treatment group the patient is randomly assigned to. It is anticipated that each surgeon will treat roughly the same number of patients.

The study will enrol a total of 243 patients to be treated by nine orthopaedic surgeons at Western Health; this accounts for approximately 81 patients per operation type.

#### Sample size

Sample size is computed on the basis of our primary hypothesis that at least one of the three surgical methods will be superior to one other surgical method for total hip replacement, as measured by the change from pre-operation to 24 months post-procedure in the overall Oxford Hip Score. The minimal clinically important difference is at least five points and the best estimate for the standard deviation is nine points [25–28]. To control for the Type I error rate due to three treatment comparisons, a Holm–Bonferroni multiplicity adjustment will be used (i.e., a two-sided significance level of 0.0167 instead of 0.05). Using a two-sample *t* test, this leads to a sample size of 70 patients per operation type to obtain 80% power. This sample size needs to be increased because of two factors. Each surgeon will only perform one surgical method and as a result each patient cannot be assumed to generate independent observations because patients are clustered by surgeon. As a result, the sample size is inflated by 2.2% (i.e., a factor [1 + (*m* − 1) × intracluster correlation coefficient]) to achieve the variance that would have been anticipated had there been no clustering, assuming that each surgeon will treat 23 (*m* = 23) patients and the intracluster correlation coefficient is 0.001 [29, 30]. In addition, a drop-out rate of 10%, which includes a mortality of 2%, is anticipated at 24 months post-procedure [31], leading to an additional 11.1% increase in sample size.

#### Data analysis

Baseline data consisting of demographic data (e.g., age, sex, diabetes, smoking status), medical history and the Kellgren–Lawrence grading will be summarised by operation type and overall and baseline imbalances will be explored.

The analysis of Oxford Hip Score and visual analogue scale will adopt the intention-to-treat approach, analysing all patients according to their randomly assigned operation type. The primary parameter Oxford Hip Score assesses 12 items on symptoms and functional status; the sum of the scores for the 12 items yields an overall score with range 0 to 48, where higher overall Oxford Hip Scores represent better outcomes.

The change from pre-operation to post-procedure in overall Oxford Hip Score will be analysed using a mixed-model repeated measures analysis, including as fixed in the model the categorical effects operation type, visit and operation type by visit interaction, and the stratification factor Kellgren–Lawrence grade, as well as the fixed continuous covariates pre-operation overall Oxford Hip Score and pre-operation overall Oxford Hip Score by visit interaction.

- To account for repeated measurements within a patient, an unstructured (co)variance structure will be used to model within-patient errors, whereby a patient is nested by surgeon.
- To account for potential clustering of the outcome due to a single surgeon using only one operation type, robust standard errors will be used. This primary model will include all patients with a pre-operation and at least one post-procedure overall Oxford Hip Score.

The primary hypothesis will be examined by three contrasts evaluating the change from pre-operation to 24 months post-procedure in overall Oxford Hip Score for the posterior versus Hardinge approach, the posterior versus anterior approach, and the Hardinge versus anterior approach using an alpha level of 1.67% for each contrast to allow for the three comparisons. The primary hypothesis will be rejected if at least one of the three comparisons reaches statistical significance using the two-sided Holm–Bonferroni-adjusted alpha level. An estimate and corresponding two-sided multiplicity-adjusted 98.33% confidence interval will be provided for each comparison. This analysis method uses a model-based approach to handle missing data providing valid inference if the missing data mechanism is ignorable (i.e., missing completely at random or missing at random). A range of sensitivity analyses to examine the robustness of the primary model results will be performed to explore the impact of the results of different missing data techniques under the same and under different missingness assumptions.

The primary model will be extended to include potential confounders, such as age, sex, diabetes or smoking status, to obtain adjusted treatment effect estimates. To investigate whether there is a different treatment effect between the subgroups defined by the Kellgren–Lawrence grade, appropriate interaction tests will be performed. The key secondary parameter measuring self-reported pain will be measured using a visual analogue scale with range 0 to 100%, where lower values represent a better outcome, and will be analysed similarly to the primary parameter.

Complications will be reported for all randomised patients who had their surgery according to the operation type they actually received. The number and percentage of patients with dislocation, infection, intraoperative and periprosthetic fractures during the entire post-procedure trial period will be reported by operation type and operation types will be compared using a modified Poisson model within the model operation type and the subgroups defined by the Kellgren–Lawrence grade whilst accounting for clustering by surgeon via robust standard errors. Residual regional pain measured using a visual analogue scale will be investigated similarly to the key secondary parameter. A two-sided alpha level of 5% will be used throughout, except for the primary parameter.

## Discussion

The relative advantages of specific surgical approaches in total hip arthroplasty are controversial, with conflicting published medical literature reporting on intraoperative blood loss [32, 33], post-operative pain [34], recovery time [35, 36], wound cosmesis [37], length of hospital stay and complication rate [38]. Also, much of the available literature are Level 4 case series with confounding factors, such as patient selection, patient and family education, rehabilitation protocol and perioperative pain [39, 40].

Restrepo *et al*. [41], in a randomised control trial, demonstrated some early short term benefits of the anterior approach over the Hardinge approach. There have been both retrospective and prospective cohort studies comparing lateral and posterior approaches [42–44], but no randomised controlled trials. A 2006 Cochrane systematic review demonstrated that neither the Hardinge nor the posterior approach is superior [45] in terms of pain, nerve injury, dislocation rates and muscle weakness.

A recent (2015) review article by Petis *et al*. [46] in the *Canadian Journal of Surgery*, concluded that

*“High-quality clinical comparisons among the approaches are lacking in the literature; therefore, surgeon preference is likely more a function of training and anecdotal success. […] Future research should elicit the long-term implications of surgical approach on clinical outcomes, restoration of function (i.e., gait analysis) and health economics.”* [46]

The Oxford Hip Score was developed in 1996 for assessing outcomes of pain and function after hip replacement surgery for use in clinical trials [47]. The validity and reliability of this score was established in the index study and subsequently examined and confirmed in a number of independent studies [26, 48, 49]. This score has even been shown to be a valid predictor of early total hip arthroplasty revision [50].

The visual analogue scale has been established as the most sensitive and reproducible way of self-reporting pain for surgical patients. Its validity for total hip arthroplasty has been established [51]; it has been shown to be equally as accurate as more complex scoring systems, such as the Western Ontario and McMaster Universities Osteoarthritis Index, 36-Item Short Form Survey and EQ-5D.

Whilst national joint registries are now collecting data regarding surgical approach and patient-reported outcome measures, to our knowledge, there is no high-level study directly comparing the three aforementioned approaches and there is a paucity of follow-up data regarding the approaches for total hip arthroplasty [52]. This study aims to address this current void in the orthopaedic literature.

## Additional file

Additional file 1: SPIRIT checklist. (DOC 121 kb)
